# The Influence of Adjuvant Type on the Immunogenicity of RBD/N Cocktail Antigens as a Vaccine Candidate against SARS-CoV-2 Virus

**DOI:** 10.1128/spectrum.02564-22

**Published:** 2023-05-18

**Authors:** Gabriela Brzuska, Marta Zimna, Klaudia Baranska, Boguslaw Szewczyk, Petra Strakova, Daniel Ruzek, Ewelina Krol

**Affiliations:** a Department of Recombinant Vaccines, Intercollegiate Faculty of Biotechnology, University of Gdansk and Medical University of Gdansk, Gdansk, Poland; b Laboratory of Emerging Viral Infections, Veterinary Research Institute, Brno, Czech Republic; c Institute of Parasitology, Biology Centre of the Czech Academy of Sciences, Ceske Budejovice, Czech Republic; d Department of Experimental Biology, Faculty of Science, Masaryk University, Brno, Czech Republic; Pontificia Universidad Católica de Chile

**Keywords:** SARS-CoV-2, adjuvants, antigen specificity, vaccines

## Abstract

The emerging virus SARS-CoV-2 (Severe Acute Respiratory Syndrome Coronavirus 2 virus), agent of COVID-19, appeared in December 2019 in Wuhan, China, and became a serious threat to global health and public safety. Many COVID-19 vaccines have been approved and licensed around the world. Most of the developed vaccines include S protein and induce an antibody-based immune response. Additionally, T-cell response to the SARS-CoV-2 antigens could be beneficial for combating the infection. The type of immune response is greatly dependent not only on the antigen, but also on adjuvants used in vaccine formulation. Here, we compared the effect of four different adjuvants (AddaS03, Alhydrogel/MPLA, Alhydrogel/ODN2395, Quil A) on the immunogenicity of a mixture of recombinant RBD and N SARS-CoV-2 proteins. We have analyzed the antibody and T-cell response specific to RBD and N proteins and assessed the impact of adjuvants on virus neutralization. Our results clearly indicated that Alhydrogel/MPLA and Alhydrogel/ODN2395 adjuvants elicited the higher titers of specific and cross-reactive antibodies to S protein variants from various SARS-CoV-2 and SARS-CoV-1 strains. Moreover, Alhydrogel/ODN2395 stimulated high cellular response to both antigens, as assessed by IFN-γ production. Importantly, sera collected from mice immunized with RBD/N cocktail in combination with these adjuvants exhibited neutralizing activity against the authentic SARS-CoV-2 virus as well as particles pseudotyped with S protein from various virus variants. The results from our study demonstrate the immunogenic potential of RBD and N antigens and point out the importance of adjuvants selection in vaccine formulation in order to enhance the immunological response.

**IMPORTANCE** Although several COVID-19 vaccines have been approved worldwide, continuous emergence of new SARS-CoV-2 variants calls for new efficient vaccines against them, providing long-lasting immunity. As the immune response after vaccination is dependent not only on antigen used, but also on other vaccine components, e.g., adjuvants, the purpose of this work was to study the effect of different adjuvants on the immunogenicity of RBD/N SARS-CoV-2 cocktail proteins. In this work, it has been shown that immunization with both antigens plus the different adjuvants studied elicited higher Th1 and Th2 responses against RBD and N, which contributed to higher neutralization of the virus. The obtained results can be used for design of new vaccines, not only against SARS-CoV-2, but also against other important viral pathogens.

## INTRODUCTION

Severe acute respiratory syndrome coronavirus 2 (SARS-CoV-2), which was first identified in Wuhan, China, in late December 2019, is a highly transmissible and pathogenic coronavirus, being the causative agent of COVID-19. The virus is a causative agent of a pandemic acute respiratory coronavirus disease 2019 (COVID-19). The infection with SARS-CoV-2 is often associated with a respiratory illness accompanied by fever, cough, shortness of breath, and muscle ache. In some cases, especially in elder patients, it can progress to severe disease resulting in multiorgan disfunction or even death (1). Since 11 March 2020, when WHO officially characterized the global COVID-19 outbreak as a pandemic, more than 525 million cases of COVID-19 have been confirmed globally, including over 6.2 million deaths. The virus is continuously spreading worldwide and has become a serious threat to global health and public safety.

As of 28.05.2022, 38 vaccines based on different platforms have been approved in 197 countries (https://covid19.trackvaccines.org/vaccines/approved/). These platforms include protein subunit, virus-like particles, DNA, mRNA, nonreplicating viral vectors, and inactivated virus. Many other vaccine candidates are being tested in clinical trials. All vaccines developed until now are able to induce an immune response against the spike protein (S), which is the main structural glycoprotein of SARS-CoV-2, anchored in its envelope. S protein plays a relevant role in the viral life cycle mediating the entry process to host cells, through interaction with receptors and membrane fusion. S protein is composed of two functional subunits: receptor-binding aminoterminal S1 subdomain and a carboxyl-terminal S2 stalk subdomain responsible for virus-host membrane fusion. The part of the S protein mainly responsible for the binding to receptors is called a receptor binding domain (RBD), located in the S1 domain of this protein (2). Several receptors and entry factors interacting with RBD have been discovered so far, including most importantly human angiotensin-converting enzyme 2 (hACE2) and others: transmembrane serine protease 2 (TMPRSS2), neuropilin-1, asialoglycoprotein receptor 1 (ASGR1), and kringle containing transmembrane protein 1 (KREMEN1) (3, 4). Some of them bind S protein not only in the RBD region, but also in other domains (5). Immune response elicited during vaccination with S protein is mainly based on the antibody response, due to the induction of neutralizing antibodies, mostly against RBD. Immunization of animal models with RBD antigen in combination with aluminum hydroxide induced protective immune response, with domination of antibody response and some level of cellular responses, including memory T-cells (6). This study has also shown higher immunogenic potential of RBD compared to S1 domain and even full S protein.

One of the approved vaccine platforms against SARS-CoV-2 are protein subunit vaccines, which are a combination of antigen and adjuvant. Two examples of such vaccines are Nuvaxovid (developed by Novavax)-stabilized S trimer with Matrix-M adjuvant (saponin-based adjuvant) and Zifivax (developed by Anhui Zhifei Longcom)-tandem-repeat RBD dimer with aluminum hydroxide. Both vaccines were shown to induce Th1/Th2 balanced immune response with a high level of neutralizing antibodies (7–10). However, these vaccines are solely based on the S protein/RBD, and given the SARS-CoV-2 mutation rate, especially in these regions, and the emergence of novel variants, it is of great importance to study other types of antigens that are immunogenic and highly conserved among SARS-CoV-2 strains. An example of such a protein is nucleocapsid protein N, which is a structural protein that binds to the genome forming the nucleocapsid. The N protein shares ~85% amino acid sequence identity between SARS-CoV-2 and SARS-CoV-1, in contrast to S protein- ~76% amino acid sequence identity. For SARS-CoV-1 virus, it has been reported that N protein could induce T-cell responses in mice; however, prior immunization with vaccinia virus expressing N before virus infection resulted in enhancement of infection due to Th2/Th1 imbalance in BALB/c mice (11, 12). For SARS-CoV-2 it was shown that N is able to induce T-cell responses in combination with RBD and Alhydrogel adjuvant (13). Moreover, Hong et al. showed that addition of N protein to a vaccine that uses RBD as antigen and Alhydrogel as adjuvant promoted not only an increase in the neutralizing antibody and T cell response, but also provided faster virus clearance in a challenge study in nonhuman primates (14). However, in both studies alum-based adjuvants were used, which are known to induce Th2-biased immune response. An ideal vaccine candidate for viruses should be able to increase a strong neutralizing antibody response, along with a balanced Th1/Th2 response, which should confer to the long-lasting immunity. The type of immune response is also greatly dependent on the adjuvant used in the vaccine formulation. Thus, rationale design of a vaccine formulation, taking into consideration the proper combination of antigen and adjuvant, is of high importance for protection against viruses and the ensuring safety profile. Several groups have studied the influence of various adjuvants but solely on the immunogenicity of recombinant RBD or S antigens (15–17).

Therefore, in this study, we have analyzed the influence of various adjuvants (AddaS03, Alhydrogel/MPLA Synthetic VacciGrade, Alhydrogel/ODN 2395 VacciGrade, and Quil-A) inducing Th1- and Th2-type immune response on the immunogenicity of a mixture of recombinant RBD and N SARS-CoV-2 proteins. These adjuvants were selected due to their ability to induce both antibody and T-cell responses. AddaS03 is an analogue of AS03 adjuvant (GSK) and has been used in combination with recombinant S protein, with positive results in phase 2 clinical trials (18). Alhydrogel/MPLA system is similar to the AS04 adjuvants (GSK) used in the licensed vaccine against HPV and HBV. Alhydrogel/ODN 2395 has been used in preclinical studies with RBD and has shown strong adjuvant properties (19). Moreover, another CpG (class B 1018) is also used in Heplisav-B vaccine against HBV. Quil A is an analogue of the QS21 saponin fraction, which is an active ingredient in Matrix M adjuvant used in combination with recombinant S protein in the Novavax vaccine (Nuvaxovid). We have analyzed the antibody and T-cell response and assessed the impact of adjuvants on virus neutralization properties of sera from immunized mice. Furthermore, we have analyzed cross-reactivity of mouse sera with a panel of coronavirus S proteins.

## RESULTS

**FIG 1:**
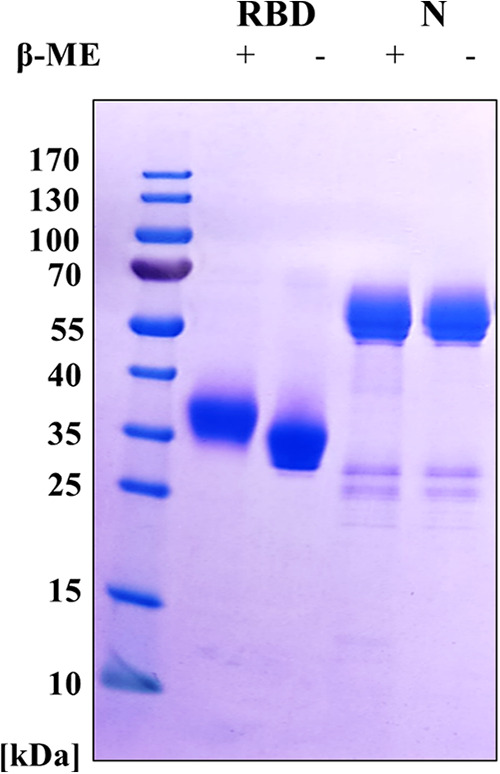
Coomassie brilliant blue staining of RBD and N recombinant proteins in reducing and nonreducing conditions. 6 μg of RBD or N recombinant proteins were resolved in SDS-PAGE electrophoresis in reducing conditions (treated with β-mercaptoethanol, β-ME; β-ME +) or in nonreducing conditions and then stained with Coomassie brilliant blue solution.

The goal of this study was to analyze the immunogenicity of RBD/N cocktail antigens in combination with various adjuvants. As the role of adjuvants on the immunogenicity of antigens was our main target, we decided to purchase recombinant glycoprotein RBD and protein N derived from Wuhan strain from Miltenyi Biotec, due to the high purity of proteins. Both proteins containing His tag and AviTag were expressed in HEK293 cells. Before immunological studies, the quality of antigens was assessed using SDS-PAGE electrophoresis in reducing and nonreducing state (Fig. 1). We observed a high purity in the RBD protein, together with the presence of monomeric forms (~35 kDa). N protein was less pure than RBD and not only the monomeric form was detected.

**FIG 2:**
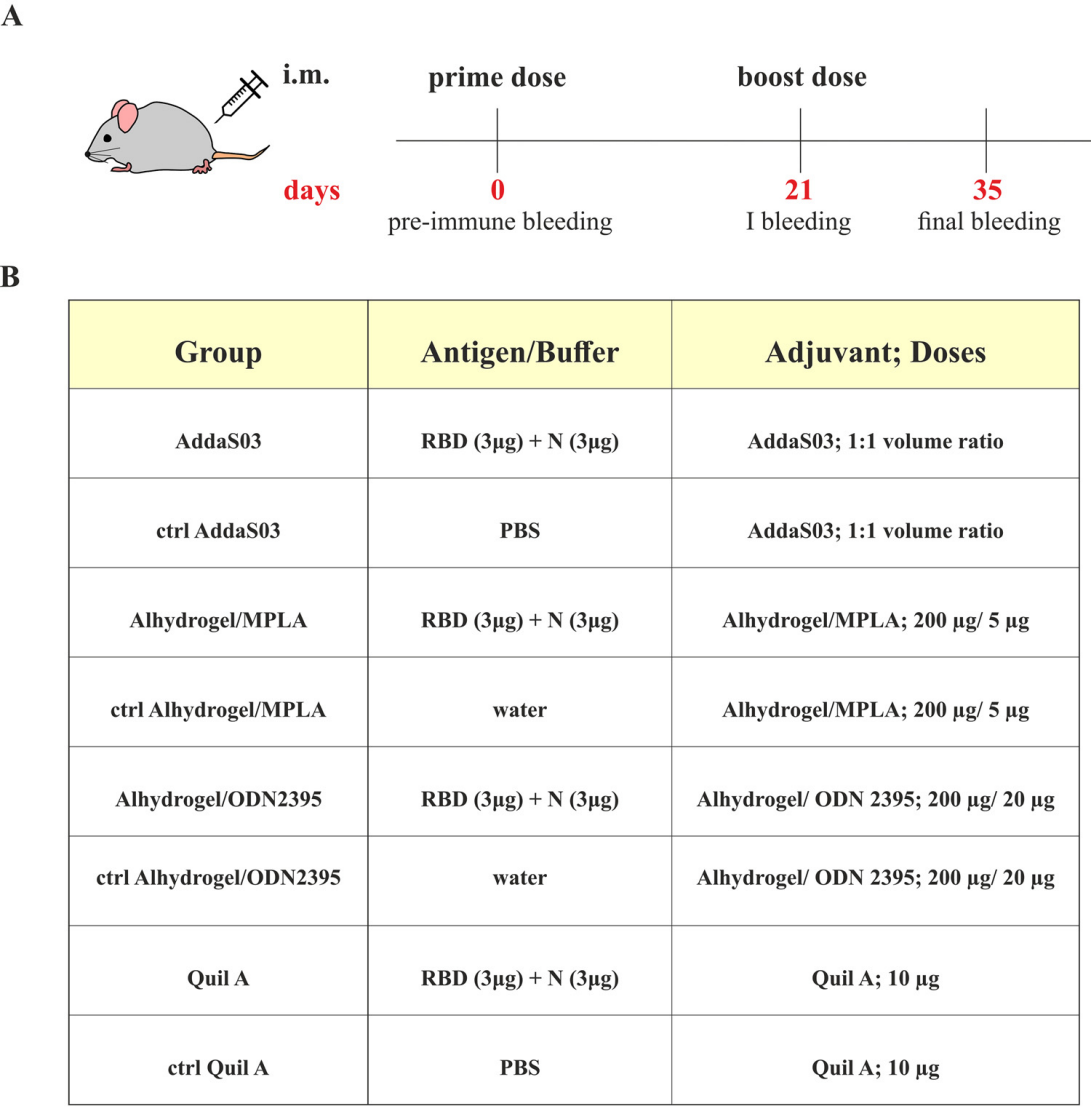
Timeline of immunization and study design. (A) schematic illustration of immunization regimen. (B) the immunization dosing for each BALB/c mice group (*n* = 6 for analyzed groups, *n* = 3 for control groups).

Next, we performed immunological studies in a mouse model with the cocktail of RBD and N antigens. Four groups of female BALB/c mice were immunized in a prime-boost regimen (3 weeks interval) with the same doses of antigens—RBD/N and various adjuvants via intramuscular route (Fig. 2). Four different adjuvant systems were used: AddaS03, Alhydrogel/MPLA Synthetic VacciGrade, Alhydrogel/ODN 2395 VacciGrade, and Quil-A (Fig. 2 B). AddaS03 is an analogue of AS03 adjuvant (GSK), composed of α-tocopherol, squalene, and polysorbate 80 in an oil-in-water emulsion, able to induce Th1/Th2/Th17 immune responses (20). Next, two adjuvants systems are based on aluminum hydroxide, which induces a Th2 type response, but differ in the addition of pattern recognition receptors (PRR) ligands: synthetic monophosphoryl lipid A (MPLA)-TLR4 agonist and class C CpG (ODN2395)—both human and mice TLR9 agonist. Both PRR ligands were shown to contribute to a Th1 type response (21–23). The fourth adjuvant used is a water-extractable fraction of saponins from the South American tree *Quillaja saponaria* Molina, with the ability of inducing Th1/Th2 balanced response with strong cytotoxic CD8^+^ lymphocyte responses (24, 25). As negative-control groups, mice were immunized with water or PBS mixed with respective adjuvants. After 2 weeks a boost dose T-cell and antibody responses were evaluated.

**FIG 3:**
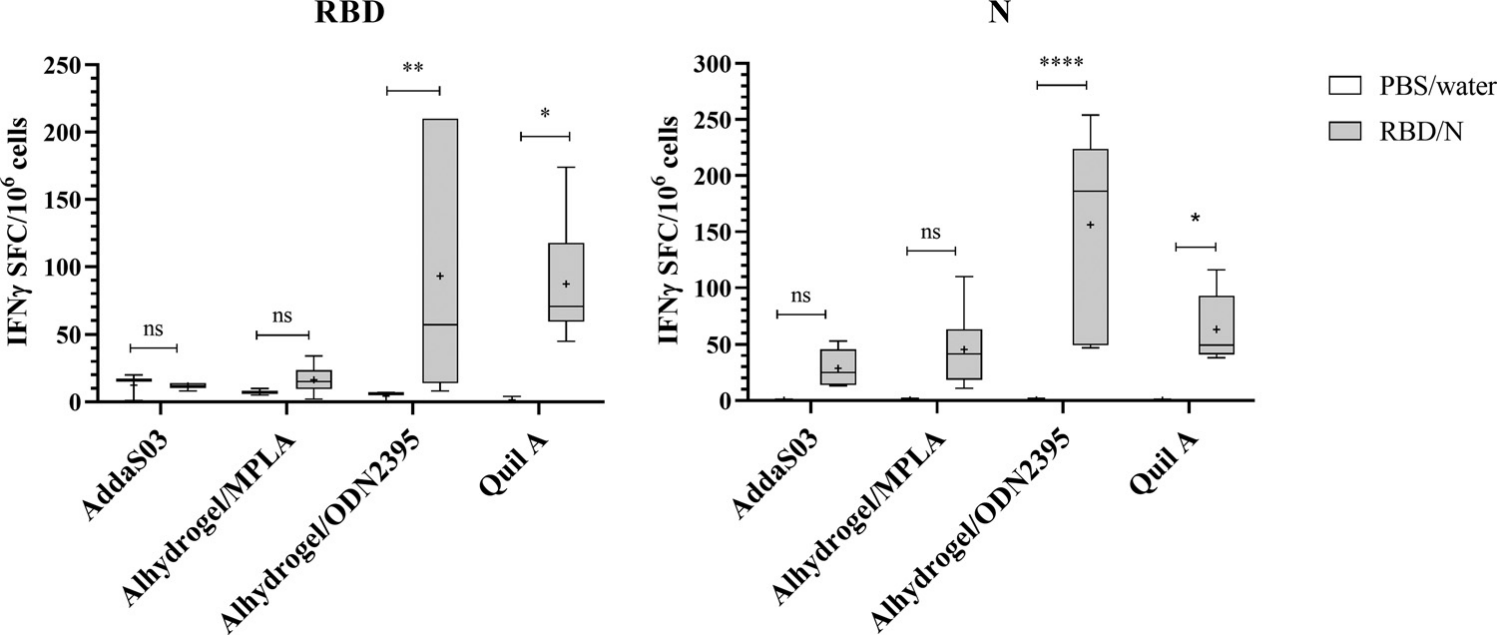
T-cell responses to RBD and N peptides measured by IFN-γ ELIspot. Box plot diagrams of IFN-γ spot-forming cells (SFC) per 10^6^ mouse splenocytes stimulated with: RBD (left panel) or N (right panel) peptides. Splenocytes from six immunized BALB/c mice per group were stimulated with pools of 15-mer overlapping peptides covering the RBD fragment or N protein sequence (1 μg/mL/peptide). The box region is the 25 to 75th percentile and whiskers are min. and max. values; the horizontal line indicates the median value and ^+^ indicates mean value. Statistical significance between immunized groups was determined using one-way ANOVA with Fisher's LSD test. *P* < 0.05: *, *P* < 0.01: **.

T-cell response was analyzed by an enzyme-linked immune spot assay (ELIspot) detecting gamma interferon (IFN-γ) levels in isolated mouse splenocytes. For the induction of IFN-γ production, splenocytes were stimulated with pools of peptides covering RBD region or N protein. Overall, as expected, RBD antigen induced slightly weaker T-cell response than N protein (Fig. 3). However, differences in the IFN-γ secreting cells were observed between vaccinated groups. AddaS03 and Alhydrogel/MPLA did not activate (for RBD) or weakly activated (for N) the production of IFN-γ, compared to the other two groups. High variances were observed in the number of IFN-γ secreting cells between mice immunized with RBD/N together with Alhydrogel/ODN2395 adjuvant, but still this adjuvant generated the strongest mean T-cell response to N (Fig. 3 right panel). Moreover, both adjuvants (Alhydrogel/ODN2395 and Quil A) were capable of induction of the highest T-cell response to RBD among all groups (Fig. 3 left panel).

**FIG 4:**
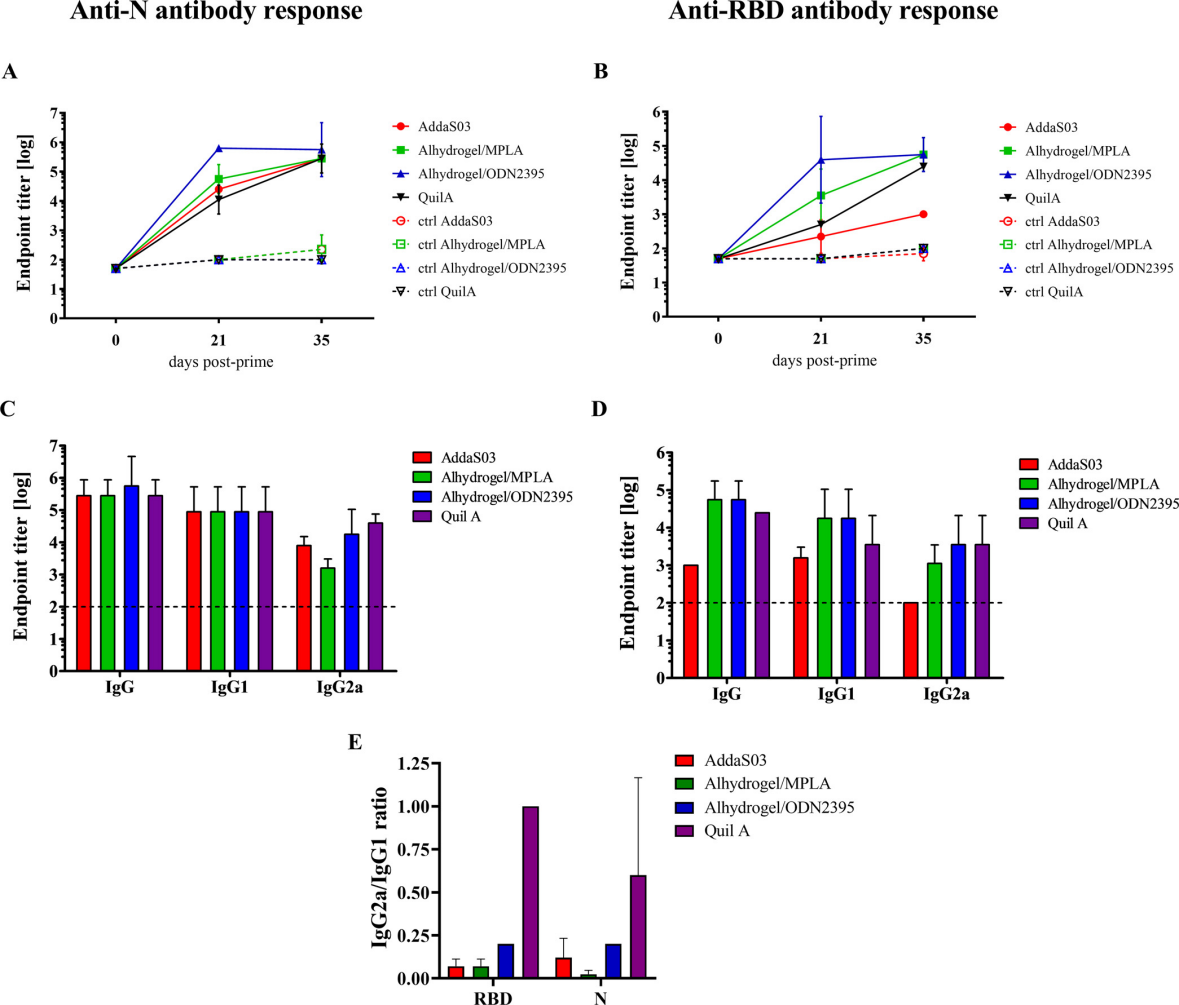
Anti-N and anti-RBD antibody response—IgG, IgG1, and IgG2a analysis in immunized mouse sera. Diagrams of anti-N (A) and anti-RBD (B) mean IgG antibody endpoint titers in the pooled preimmune and immune mouse sera. Diagrams of N-specific (C) and RBD-specific (D) IgG, IgG1, and IgG2a endpoint titers in mouse sera collected on the 35th day postprime. (E) Graph presenting anti-N and anti-RBD IgG2a/IgG1 mean endpoint titers ratio values X. The dashed lines in B and D represent the baseline of the assay-mean titer of control groups.

Next, the antibody response to RBD and N antigens was examined in the preimmune sera and in the sera collected after each immunization in order to evaluate the kinetics of antibodies generation in vaccinated animals (Fig. 4). The IgG titers were measured using ELISA on plates coated with recombinant RBD or N antigens. In almost all groups, the boost dose increased the level of anti-N and anti-RBD IgGs. Only in the Alhydrogel/ODN2395 group, the mean anti-N and-RBD antibody titers were the highest (4 and 3 logs higher than control groups, respectively) after first vaccination and remained on the same level after the boost dose. In other groups, this level of anti-N IgGs was reached after a second dose. On the contrary, titers of anti-RBD IgG antibodies were more diverse. The most potent antibody response to RBD was induced by Alhydrogel/MPLA and Alhydrogel/ODN2395 adjuvant combinations. In the Quil A group, the anti-RBD IgGs level strongly increased after a boost dose. In the AddaS03 group, even after the boost dose, the anti-RBD IgGs level was the lowest—only 10-fold above the control groups. Moreover, the titers of IgG1 and IgG2a, indicators of the Th2 and Th1 type immune responses, respectively, were determined in mouse sera collected on the 35th day postprime (Fig. 4C and D). The titers were used for calculation of IgG2a/IgG1 ratio for visualization of antibody response polarization (Fig. 4E). The level of IgG1 antibody to N antigen was the same in all tested groups in contrast to IgG2a. The most balanced response to both RBD and N was induced by Quil A adjuvant. The lowest titer of anti-N IgG2a antibody was observed in the Alhydrogel/MPLA group and highest level was induced byQuil A. Although both of these adjuvant systems induced Th2-skewed immune response, the Alhydrogel/ODN2395 promoted slightly higher Th1 immune response, given also the higher IFN-γ production (Fig. 3). AddaS03 adjuvant also activated Th2-polarized response.

**FIG 5:**
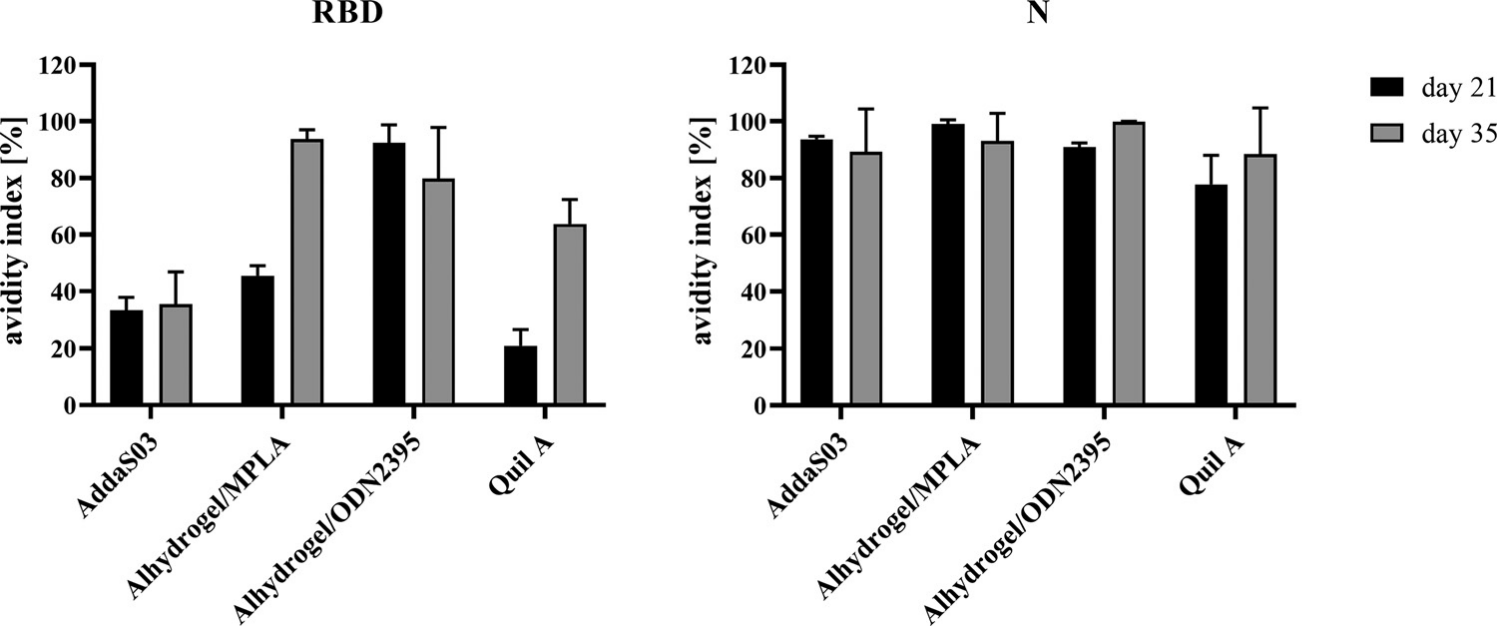
The avidity of IgG antibodies directed toward RBD and N antigens. The avidity ELISA was performed with the use of KSCN solution to measure the binding strength of IgGs in the sera from the 21st and 35th day postprime to RBD (left panel) or N (right panel) antigens. The avidity index is the ratio of the mean absorbance value from the KSCN treated sample to the nontreated sample and expressed as a percentage.

Furthermore, the avidity of IgG antibodies directed toward RBD and N antigens was evaluated to further characterize the immune response. We performed an avidity ELISA using mouse sera collected from day 21 and 35 (Fig. 5). Anti-N antibodies exhibited very high avidity from the first dose, regardless of the adjuvant used. However, for the RBD antigen, only the Alhydrogel/ODN2395 adjuvant was able to induce high avidity IgGs from the first dose of vaccination. In the Alhydrogel/MPLA and Quil A groups, the avidity of antibodies was increased after the boost dose. AddaS03 adjuvant was not able to induce high avidity antibodies, even after boosting.

**FIG 6:**
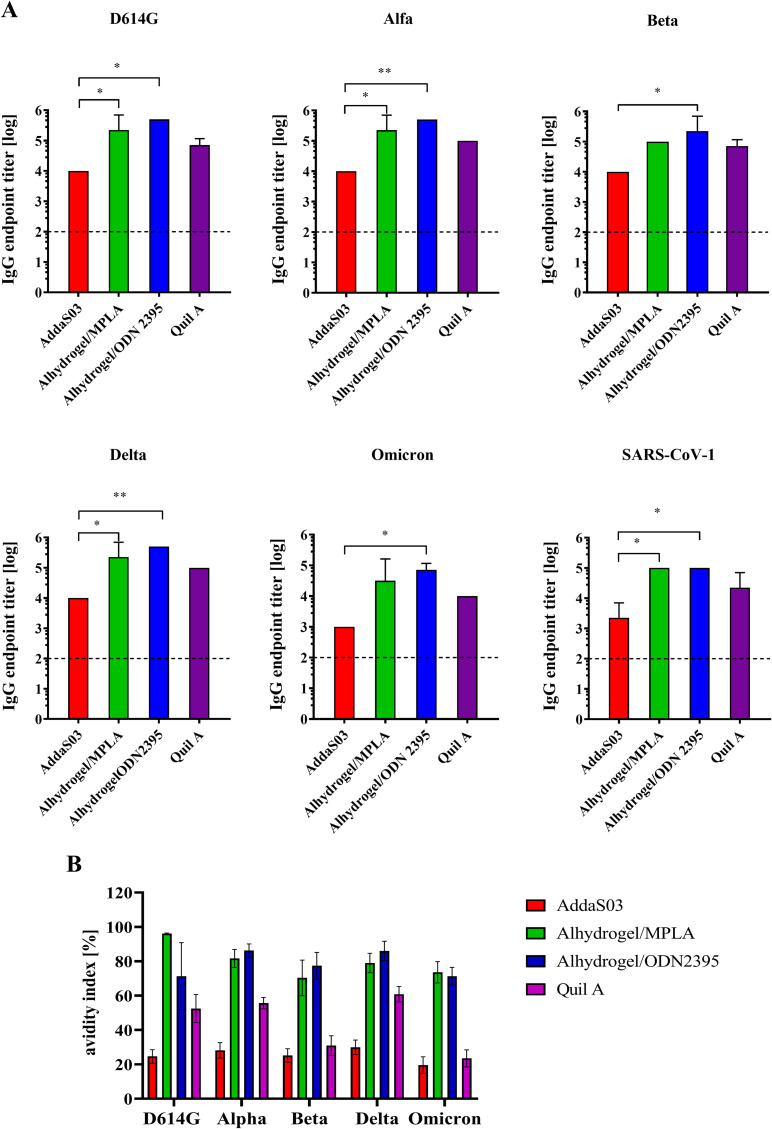
Cross-reactive IgG responses to various coronavirus Spike antigens. Diagrams of mean cross-reactive IgG antibody endpoint titers to variants of S protein: Wuhan strain with D614G mutation, Alfa, Beta, Delta, Omicron, and extracellular domain of S from SARS-CoV-1 in the immune mouse sera (day 35 postprime) (A). The IgGs were titrated using ELISA on plates coated with respective S protein variants. The dashed line represents the baseline of the assay-mean titer of control groups. Statistical significance was calculated using one-way ANOVA with Tukey’s multiple comparison. *P* < 0.05: *, *P* < 0.01: **. The avidity of IgG antibodies directed toward various SARS-CoV-2 S antigens (B). The avidity ELISA was performed with the use of KSCN solution to measure the binding strength of IgGs in the sera from 35th day postprime to trimeric S derived from several variants of SARS-CoV-2 (D614G, Alpha, Beta, Delta, Omicron). The avidity index is the ratio of the mean absorbance value of the KSCN-treated sample to the nontreated sample and expressed as a percentage.

Next, we analyzed the cross-reactivity of mouse sera with a trimeric form of S protein from 5 variants of SARS-CoV-2: Wuhan strain with D614G mutation, Alfa (B.1.1.7), Beta (B.1.351), Delta (B.1.617.2), Omicron (B.1.1.529) and extracellular domain of S from SARS-CoV-1 (Fig. 6A). All tested sera were cross-reactive to S variants. The titers of IgGs cross-reactive to S antigens of D614G variant, Alfa, Beta, and Delta were on similar level in each group, except a slightly lower immune response to Omicron variant and SARS-CoV-1. However, the elicited endpoint IgGs titers in mouse sera varied in respect to the used adjuvant. The highest IgGs level to all S antigens were induced by Alhydrogel/ODN2395 and slightly lower by Alhydrogel/MPLA adjuvant system. The lowest titers were detected in a group immunized with AddaS03 adjuvant. Quil A adjuvant gave medium immune response. The avidity of cross-reactive IgGs directed to SARS-CoV-2 S trimer variants (D614G, Alpha, Beta, Delta, Omicron) was also determined (Fig. 6B). Similar to the results obtained with the RBD antigen, the IgGs with the highest avidity were observed in the group immunized with Alhydrogel/MPLA and Alhydrogel/ODN2395. The IgGs showed medium avidity in the Quil A immunized group, and AddaS03 did not induce high-avidity antibodies directed against any of the S protein variants.

**FIG 7:**
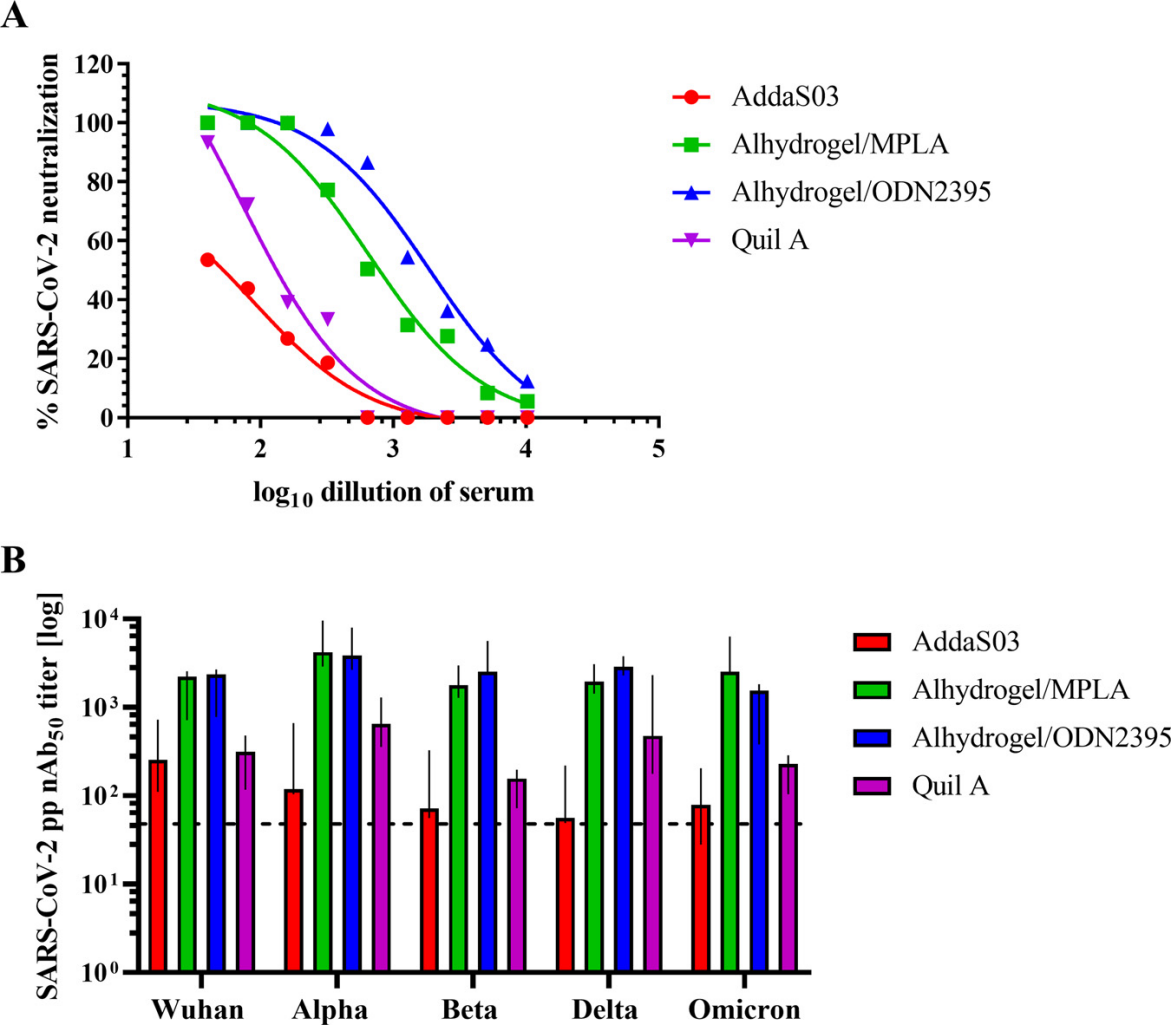
Neutralization of SARS-CoV-2 virus. The neutralizing activity of mouse sera collected on day 35 was evaluated in a plaque-reduction neutralization assay with authentic SARS-CoV-2 virus (A). Pseudoparticles neutralization assay was utilized to evaluate cross-neutralizing antibody titers (mean 50% neutralizing antibody titer [nAb_50_]) against different variants (Wuhan strain, Alpha, Beta, Delta, and Omicron variants) in A549 hACE2/TMPRSS2 cells (B). Mean nAb_50_ were calculated using four-parameter logistic curve fitting in GraphPad Prism software; whiskers are a 95% CI values. Dashed line is a mean nAb_50_ value of negative-control groups.

Finally, the influence of the adjuvant type on the SARS-CoV-2 virus neutralization by antibodies elicited by immunization with RBD/N cocktail was assessed. Two different assays were employed: plaque-reduction neutralization with SARS-CoV-2 virus and SARS-CoV-2 pseudoparticles neutralization in A549 hACE2/TMPRSS2 cells (Fig. 7). Mice immunized with Alhydrogel/MPLA and Alhydrogel/ODN2395 demonstrated the highest neutralizing activity against authentic virus. PRNT_50_ titer in Alhydrogel/MPLA was 1:655.6 (95% CI, 1:356.6-1:1226), and the highest PRNT_50_ titer was observed in the Alhydrogel/ODN2395 group, reaching 1:1875 (95% CI, 1:992.2-1:3768). The weakest neutralization was observed in the Quil A (PRNT_50_, 1:72.7; 95% CI, 1:21.3-1:171.5) and AddaS03 group A (PRNT_50_, 1:90.9; 95% CI, 1:34.7-1:193.0). The results were similar in the assay with pseudotyped particles with S protein derived from different SARS-CoV-2 variants. The highest nAb50 titers were observed in the groups immunized with Alhydrogel combined with MPLA or ODN2395 and the lowest with AddaS03, which is in agreement with cross-reactivity ELISA results and avidity index of IgGs.

Overall, the results from all the performed analyses demonstrated the superiority of the Alhydrogel combined with PRR agonists MPLA or ODN2395 over other tested adjuvants in inducing an immune response against SARS-CoV-2.

## DISCUSSION

Although, many vaccines have been licensed for the use against SARS-CoV-2 virus, still there is a need for safe and effective vaccines especially for elderly populations. Currently used anti-COVID-19 vaccines exhibit sometimes allergic reactions, which may be attributed to the high levels of generated IgG antibodies that interact with the Fcγ receptors present on the surface of some immune cells (26, 27). It is of great importance that vaccines elicit an immune response capable of neutralizing the circulating variants of concern and possible new strains so as to induce immune memory cells and actively prevent the development of disease. Furthermore, it should be borne in mind, these vaccine antigens should be easy to manufacture and not require ultracold storage conditions, and therefore subunit recombinant proteins are good candidates for vaccination antigens. Manipulation of their immunogenicity may be achieved by choosing optimum adjuvants.

Our study describes the impact of adjuvants on the immunogenicity of RBD/N cocktail antigens. The RBD domain of S protein contains fewer immunodominant CD4^+^ T-cell epitopes than the full-length protein. Despite this fact, RBD may exhibit superiority over S protein in inducing neutralizing antibodies, though addition of an adjuvant is crucial for this purpose (6). This may depend on the oligomeric state of RBD, as it was shown that dimers or trimers induced a higher level of antibodies (28), which can be further stimulated with the use of various adjuvants, e.g., alum, alum/MPLA, alum/CpG, Alum/saponins, AddaS03, or manganese nanoparticles (16, 17, 19, 29). The data from these studies suggest that inclusion of TLR agonist like MPLA or CpG next to alum has a beneficial effect on the RBD immunogenicity. As RBD is a good antigen for the induction of neutralizing antibodies, but not for the induction of cytotoxic immune response, the addition of other elements influencing this type of response to the vaccine formulation is a good strategy in combating COVID-19. An example of these elements is the N protein due to its high immunogenicity and the fact that it is more conserved among coronaviruses than S protein (30). Moreover, several studies have shown the protective effect of N protein after immunization with RBD-based antigens (13, 14, 31). The level of IFN-γ– and IL-4–producing cells, upon stimulation with RBD peptides, was increased in the group immunized with the RBD-P2/N combination compared with their level in the group immunized with RBD-P2 alone (14). Moreover, in the challenge experiment this protein provided faster clearance of the virus. Compared to our study, in all of these reports the higher dosage of RBD and N antigens was used, and in addition, two studies used alum as an adjuvant, which is a good adjuvant for inducing antibody generation, but mainly a Th2 type immune response is induced (13, 14). We hypothesized that the immunogenic potential of a low-dose RBD/N antigen cocktail could be enhanced with adjuvants that stimulate both Th1 and Th2 immune response. Therefore, in our study we tested 4 adjuvants: squalene-based oil-in-water emulsion (AddaS03), aluminum hydroxide supplemented with TLR4 agonist- monophosphoryl lipid A (Alhydrogel/MPLA) or TLR9 agonist- class C CpG, ODN2395 (Alhydrogel/ODN2395), and saponins (Quil A). All vaccine formulations were used for immunization of animals in a prime-boost regimen. Our data have shown that all formulations were able to induce specific immune response; however, the Alhydrogel/ODN2395 and Alhydrogel/MPLA adjuvants led to the highest neutralizing antibodies levels. Quil A and Alhydrogel/ODN2395 stimulated high cellular response to both RBD and N, as assessed by IFN-γ production. All adjuvants induce Th2-skewed immune response, as IgG1 levels are higher than IgG2a; however, Quil A was able to induce slightly more Th1-polarized response. However, observed Th2 profiles could be also influenced by the general Th2-polarization of immune response in BALB/c mice strain. The ideal vaccine candidate for COVID-19 should induce balanced Th1/Th2 immune response. There are reports that some human neutralizing monoclonal antibodies against S/RBD of SARS-CoV-2 may induce antibody-mediated enhancement of infection through interaction with Fcγ receptors (32, 33). Moreover, for SARS-CoV-1 it was found that prior vaccination to virus challenge in mice with N protein could contribute to immunopathology, through Th2-cell-biased responses (12). Thus, it is of vital importance that vaccine formulations stimulate not only Th2/antibody response to viral antigens, but also Th1/cytotoxic immune response in order to eliminate infected cells. Although, knowledge about enhancement of SARS-CoV-2 infection in humans is still limited and much is to be discovered, it should always be considered in the development of new vaccine formulations.

Moreover, we have shown in our study that the mice group vaccinated with Alhydrogel/ODN2395 showed a very strong production of high-avidity anti-RBD and anti-N antibodies, since first dose of vaccination, which was maintained at the same level after the boost dose, which was not observed in other groups. ODN2395 may stimulate the production of IFN-α from pDC and enhance B cell activation and proliferation (34). Used together with alum, OD2395 may modulate the adjuvant activity of alum, and may promote IFN-y response (35). Pollet et al. also used a combination of Alhydrogel and class B CpGs as an adjuvant system together with RBD antigen (36). This group also reported a strong antibody response after two vaccinations, regardless of the CpGs used in the boost dose. However, they showed enhanced cytokine production (e.g., IFN-y) after addition of CpGs in a boost dose. Sparwasser et al. demonstrated that CpG DNA could activate immature antigen-presenting DCs to professional APC (37). In addition, Klinman et al. demonstrated that CpG-adjuvanted anthrax vaccine could accelerate serum antibody response (38). Therefore, we can speculate that ODN2395 may modulate the activity of Alhydrogel, which further resulted in more balanced immune response with the production of high-avidity antibodies.

The next goal of our study was the evaluation of cross-reactivity of immune mouse sera with a panel of coronavirus S proteins. There are reports showing that current vaccines may not be efficient in generation of high levels of neutralizing antibodies against some SARS-CoV-2 variants, e.g., Omicron (39, 40). Our study has shown that Alhydrogel/ODN2395 and Alhydrogel/MPLA adjuvants provided the highest level of cross-reactive neutralizing antibodies to S protein variants from various SARS-CoV-2 strains, including the Omicron variant. Moreover, these adjuvants were also able to induce the highest level of antibodies to SARS-CoV-1. Structures of RBD region of SARS-CoV-2 and SARS-CoV-1 are highly similar, and some monoclonal antibodies targeting RBD are able to neutralize both viruses (41). Due to that, and the high conservation of N protein, such combination of antigens could have a potential as a bivalent vaccine.

Our study shows the activation of a strong antibody response directed toward S protein in groups immunized with Alhydrogel/MPLA and Alhydrogel/ODN2395. In addition, ODN2395 modulated the immunogenicity of antigens and led to strong production of high avidity IgGs and activation of a T-cell response to RBD and N peptides. It would be interesting to characterize further the mechanism of action of this adjuvant system. Moreover, Nanishi et al. demonstrated the persistence (up to 210 days) of antibody response after immunization with two doses of RBD alone in combination with alum/ODN2395 in adults and aging mouse models (19). Therefore, it would be noteworthy to analyze the durability of the anti-RBD and anti-N immune response after immunization with our combination in a follow-up study.

Together with other studies examining RBD/N combinations, our results confirm the high potency of these antigens as immunogens that can activate potent and persistent protection against novel SARS-CoV-2 variants. In addition, our study highlights the importance of the evaluation of adjuvants used in this combination. In particular, the use of Alhydrogel in combination with MPLA or ODN2395 may lead to the production of high titers of neutralizing antibodies with high avidity and may also stimulate a T-cell response in the case of ODN2395.

To sum up, the presented results suggest that formulation composed of RBD/N cocktail in combination with the optimum adjuvant system may be of high potential as vaccine candidate, targeting Th1 and Th2 immune response.

## MATERIALS AND METHODS

### Cells and viruses.

A549 hACE2/TMPRSS2, a lung carcinoma cell line overexpressing SARS-CoV-2 receptors: human ACE2 (angiotensin-converting enzyme 2), and TMPRSS2 (transmembrane serine protease 2) was obtained from InvivoGen and was used for pseudoparticles neutralization assay. Cells were cultured in Minimum Essential Medium Eagle (EMEM) with Earle's Balanced Salt Solution (EBSS) (Lonza) supplemented with 2 mM l-glutamine, 10% (vol/vol) heat-inactivated fetal bovine serum (FBS), 100 U/mL penicillin, 100 μg/mL streptomycin, puromycin (0.5 μg/mL), hygromycin (150 μg/mL).

Vero cells (ATCC CCL-81, African Green Monkey, adult kidney, epithelial) were cultured in Dulbecco's modified Eagle's medium (DMEM) supplemented with 10% newborn calf serum, 100 U/mL penicillin, 100 μg/mL streptomycin, and 1% glutamine (Sigma-Aldrich, Prague, Czech Republic).

SARS-CoV-2 (Czech strain SARS-CoV2/human/Czech Republic/951) was provided by Jan Weber, Institute of Organic Chemistry and Biochemistry, Prague, Czech Republic. This virus was passaged through Vero cells a maximum of 5 times before use.

### Proteins.

Two recombinant proteins of SARS-CoV-2: receptor binding domain (RBD) (R319 to S591 amino acid sequence, Wuhan strain, His tag and an AviTag) and nucleoprotein N (2S–419A amino acid sequence, Wuhan strain, His tag and an AviTag) were purchased from Miltenyi Biotec. Proteins were dissolved according to manufacturer’s instructions and further dialyzed against sterile water using Amicon Ultra-15 10K filters for mouse immunization experiment.

For antibody titers and analysis using ELISAs a panel of recombinant coronavirus proteins was purchased (Table 1).

**TABLE 1:** List of recombinant proteins used as antigens in ELISAs

Recombinant protein	Manufacturer
SARS-COV SPIKE S1 + S2 ECD-HIS RECOMBINANT PROTEIN (S577A, ISOLATE TOR2)	Sino Biological Europe GmbH
MERS-COV SPIKE PROTEIN (S1 + S2 ECD, AA 1-1297, HIS TAG)	Sino Biological Europe GmbH
SARS-COV-2 SPIKE S1 + S2 (D614G) TRIMER PROTEIN (ECD, HIS TAG)	Sino Biological Europe GmbH
SARS-COV-2 B.1.1.7 (ALPHA) SPIKE S1 + S2 TRIMER PROTEIN (ECD, HIS TAG)	Sino Biological Europe GmbH
SARS-COV-2 B.1.351 (BETA) SPIKE S1 + S2 TRIMER PROTEIN (ECD, HIS TAG)	Sino Biological Europe GmbH
SARS-COV-2 B.1.617.2 (DELTA) SPIKE S1 + S2 TRIMER PROTEIN (ECD, HIS TAG)	Sino Biological Europe GmbH
SARS-COV-2 B.1.1.529 (OMICRON) S1 + S2 TRIMER PROTEIN (ECD, HIS TAG)	Sino Biological Europe GmbH

### SDS-PAGE.

Analysis of recombinant proteins was performed using SDS-PAGE. Samples were run in reducing or nonreducing conditions on 10 to 20% gradient Tris-Glycine gels- Novex WedgeWell (Thermo Fisher Scientific) in Tris-Glycine SDS running buffer. After electrophoresis, the gel was stained with Coomassie brilliant blue R-250 solution.

### Immunization of mice.

Groups of 6 (3 for control groups) female BALB/c mice, 6 to 8 weeks of age, were vaccinated by intramuscular route (i.m.; hind leg thigh) with the mixture of antigens (RBD and N) and adjuvant. Mice were immunized twice (prime and boost dose) with 3 μg of each antigen—RBD and N on days 0, 21. Four different adjuvants were used: AddaS03, Alhydrogel/MPLA Synthetic VacciGrade, Alhydrogel/ODN 2395 VacciGrade, Quil-A. All adjuvants and adjuvant components were purchased from InvivoGen. Antigens were mixed with adjuvants according to manufacturer’s recommendations up to 3 h before the immunization. The total volume of one dose was 50 μL. Figure 2B presents doses of adjuvants used per one vaccination dose per mouse. The mice used as negative-control groups were immunized with each adjuvant and physiological water or phosphate-saline buffer (PBS) only. On day 35 the mice were sacrificed, and the sera and spleens were collected for immunological analysis of humoral and T-cell response. All animal experiments were conducted by an accredited institution (Tri-City Academic Laboratory Animal Centre, Medical University of Gdansk, Gdansk, Poland), in accordance with the current guidelines for animal experimentation. The protocols were approved by the Local Committee on the Ethics of Animal Experiments of the University of Science and Technology in Bydgoszcz (Permit Number: 40/2021). All surgery was performed under isoflurane anesthesia; all efforts were taken to minimize suffering.

### ELIspot assay.

T-cell response to RBD and N proteins was analyzed by ELIspot assay, detecting IFN-γ after stimulation of splenocytes according to the manufacturer’s instructions (BD Biosciences). Briefly, ELIspot 96-well plates were coated with anti-mouse IFN-γ antibody and blocked using RPMI Medium 1640 supplemented with 10% FBS, 25 mM HEPES buffer, Pen-strep antibiotic and 2 mM l-glutamine (Gibco). Splenocytes were isolated from mice by passage through 70 μm strainers followed by treatment with ACK lysing buffer (Thermo Fisher Scientific). After series of washing, splenocytes were seeded at 5 × 10^5^ cells/well and stimulated overnight at 37°C with synthetic peptides overlapping region of RBD or N proteins (PepMix SARS-CoV-2 [S-RBD], JPT Peptide Technologies GmbH; PepTivator SARS-CoV-2 Prot_N, Miltenyi Biotec). Complete medium was used as negative control. Immunospots were counted using CTL ImmunoSpot Analyzer. The mean number of spots from duplicate wells were calculated for each animal, the basal number of spots from the negative control was extracted and then adjusted to represent the mean number of spots forming cells (SFC) per 10^6^ splenocytes.

### Antibody response analysis: antibody response in time and sera titration (IgG, IgG1, IgG2a).

Antibody response was analyzed using an ELISA with various antigens listed above in Table 1. Antibody response in time was analyzed using RBD and N recombinant proteins and sera titration was performed using recombinant coronavirus spike proteins. ELISA plates were coated with antigens in concentration of 1 μg/mL. Then, plates were blocked with 3% BSA in PBS-T, washed and serially diluted (from 10^2^ to 0.5 × 10^6^) pooled mouse sera in triplicates were added to the plates and incubated 2 h at room temperature. Next, plates were washed again and secondary HRP-conjugated antibodies (0.7 μg/mL): goat anti-mouse IgG (Santa Cruz Biotechnology), goat, cross-adsorbed anti-mouse IgG1 (Thermo Fisher Scientific) or goat anti-mouse IgG2a (Thermo Fisher Scientific) were added. After washing, TMB Substrate Solution (Thermo Fisher Scientific) was used for detection of bound antibodies. The reaction was stopped with 0.5 M H_2_SO_4_ and the absorbance at 450 nm was measured using microplate reader (TECAN). Mean absorbance for each dilution of the sera was calculated and then endpoint titers of antibodies were determined as the dilution of sera which gave A_450_ signal above the baseline (twice the value of mean A_450_ of wells without sera). The analysis was done in duplicates.

### Evaluation of IgG antibodies avidity.

Serum samples were subjected to an IgGs avidity ELISA using potassium thiocyanate (KSCN) as a chaotropic agent, as previously described with minor modifications (42). Sera were diluted in PBS-T buffer to achieve the A_450_ value of ~2.2-2.8, then were added to the plate coated with respective antigen (N, RBD or recombinant coronavirus spike proteins) and blocked for 2 h in 2 replicates. After washing, wells were treated in the presence or absence of 1.5 M KSCN (200 μL/well) for 15 min. at room temperature. After washing, secondary anti-mouse IgG HRP-conjugated antibodies were added and normal ELISA protocol was continued, as described above. Avidity index was calculated as a ratio of mean absorbance value from the KSCN treated sample to the nontreated sample and expressed as a percentage.

### SARS-CoV-2 pseudotyped lentiviral particles neutralization assay.

Pseudotyped lentiviral particles with SARS-CoV-2 (Wuhan strain) were produced as published previously (43). Other variants of pseudoparticles were produced using plasmids encoding S proteins of various viral variants (Alpha B.1.1.7, Beta B.1.351, Delta B.1.617.2) -obtained from InvivoGen or Omicron (in-house produced). Particles were titrated on A549 hACE2/TMPRSS2 cell line and the dilution which gave luciferase activity of at least 1,000-fold above noninfected cells was used for neutralization assay. A549 hACE2/TMPRSS2 cells were seeded at the density of 1.2 × 10^4^ in 96-well plate on a day 0. Mouse sera were inactivated in 56°C for 30 min, serially diluted in EMEM complete medium and then added to pseudotyped lentiviral particles. Then, the mixtures were incubated in 37°C for 1 h and further added to cells for another 2 h, to allow the entry of particles to cells. After that time, EMEM complete medium supplemented with 5 μg/mL of Polybrene was added. Cells were cultured for 48h h and then luciferase activity was measured using Bright-Glo luciferase assay (Promega) as described previously. Luciferase values were measured using the microplate reader (TECAN). Neutralization antibody titers (nAb_50_) were defined as the serum dilutions that could reduce ~50% relative luminescence units compared to pseudotyped lentiviral particles infected control wells. nAb50 were calculated using a four-parameter logistic curve in GraphPad Prism software.

### Plaque reduction neutralization assay (PRNT).

Vero cells were seeded in each well of 12-well plates (approximately 5 × 10^5^ cells per well) and incubated for 2 days at 37°C and 5% CO_2_ to form a monolayer. Mouse sera were diluted 1:4 in DMEM medium and inactivated at 56°C for 30 min. Inactivated mouse sera (at dilutions of 1:40, 80, 160, 320, 640, 1280, 2560, 5120, and 10240) were incubated with the virus (100 PFU per well) for 90 min at 37°C and added to the cell monolayer in a total volume of 500 μL. After 3 h of incubation at 37°C and 5% CO_2_, the wells were washed with phosphate-buffered saline and fresh medium containing 4% (wt/vol) carboxymethylcellulose was added to the wells. After 4 days of incubation at 37°C and 5% CO_2_, cell monolayers were stained with naphthol blue black dye and the number of plaques was counted. The percentage reduction in plaques was determined for each test sample at each dilution compared to control wells (virus without sera). PRNT_50_ titers were calculated based on 50% reduction in plaque counts.

### Statistical analysis.

All statistical analyses were done using GraphPad Prism 5 software. Statistical significance was determined using a one-way ANOVA with Fisher's LSD or Tukey’s multiple comparison.

### Data availability.

The data generated and/or analyzed during this study are available from the corresponding author upon reasonable request.
